# Efficacy and safety of i.v. sodium benzoate in urea cycle disorders: a multicentre retrospective study

**DOI:** 10.1186/s13023-016-0513-0

**Published:** 2016-09-23

**Authors:** Marie-Caroline Husson, Manuel Schiff, Alain Fouilhoux, Aline Cano, Dries Dobbelaere, Anais Brassier, Karine Mention, Jean-Baptiste Arnoux, François Feillet, Brigitte Chabrol, Nathalie Guffon, Caroline Elie, Pascale de Lonlay

**Affiliations:** 1 Etablissement Pharmaceutique des Hôpitaux de Paris, AGEPS, 7 rue du Fer à Moulin, Paris, 75005 France; 2 Centre de Référence des Maladies Héréditaires du Métabolisme, Hôpital Robert-Debré, AP-HP, Paris, France; 3 Reference Centre for Inherited Metabolic Disorders, Hôpital Femme Mère-Enfant, Lyon, France; 4 Centre de Référence des Maladies Héréditaires du Métabolisme, CHU Timone Enfants, Marseille, France; 5 Centre de Référence des Maladies Héréditaires du Métabolisme, Hôpital Jeanne-de-Flandre, CHRU Lille, Lille, France; 6 Reference Centre for Inherited Metabolic Disorders (MaMEA), Hôpital Necker-Enfants Malades, Institut Imagine, Université Paris Descartes, 149 rue de Sèvres, 75743, Paris, Cedex 15 France; 7 Centre de Référence des Maladies Héréditaires du Métabolisme, CHU de Nancy, Hôpital Brabois Enfants, Vandoeuvre-les-Nancy, France; 8 URC/CIC Paris Centre, Hôpital Universitaire Necker-Enfants Malades, 149 rue de Sèvres, 75015 Paris, France

**Keywords:** Clinical trials, Hyperammonemia, Systematic review, Urea cycle disorders, Sodium benzoate

## Abstract

**Background:**

The efficacy and safety of intra-venous (i.v.) sodium benzoate for treating acute episodes of hyperammonemia in urea cycle enzyme disorders (UCD) is well known. However, published data do not provide a clear picture of the benefits and risks of this drug. We report a retrospective multicentre study on the use of i.v. sodium benzoate in patients treated for UCD between 2000 and 2010 in the 6 French reference centres for metabolic diseases.

**Results:**

Sixty-one patients with UCDs - 22 ornithine transcarbamylase (20 confirmed, 2 suspected), 18 arginino-succinate synthetase, 15 carbamoyl phosphate synthetase, 3 arginosuccinate lyase, 1 arginase deficiency, 1 N-acetylglutamate synthetase, 1 HHH syndrome - required i.v. sodium benzoate over the course of 95 acute episodes (NH3 > 100 μmol/L or high-risk situations, i.e., gastroenteritis, surgery). Forty out of 61 patients experienced only one episode of decompensation (neonatal coma, 68.6 %). The most frequent cause of late decompensation was infection (55.5 %). A loading dose of i.v. sodium benzoate (median 250 mg/kg over 2 h) was administered for 41/95 acute episodes. The median maintenance dose was 246.1 mg/kg/day, administered via peripheral venous infusion in all cases except one via a central line. The total median duration of i.v. sodium benzoate treatment per episode was 2 days (0–13 days). The median durations of hospitalization in intensive care and metabolic units were 4 days (0–17 days) and 10 days (0–70 days), respectively. Eight patients died during the neonatal coma (*n =* 6) or surgery (*n =* 2). The median plasma ammonium level before treatment was 245.5 μmol/L (20.0–2274.0 μmol/L); it decreased to 40.0 μmol/L in patients who were alive (13.0–181.0 μmol/L) at the end of treatment with i.v. sodium benzoate. A decrease in ammonium level to ≤ 100 μmol/L was obtained in 92.8 % of episodes (64/69 of the episodes recorded for the 53 surviving patients). Five patients required another treatment for hyperammonemia (sodium phenylacetate + sodium benzoate, haemofiltration). Eighteen side effects were reported related to the i.v. infusion (local diffusion, oedema).

**Conclusion:**

This 10-year retrospective study shows that i.v. sodium benzoate associated with an emergency regimen is an effective and safe treatment for acute episodes of UCD.

## Background

The urea cycle is the single metabolic pathway that eliminates circulating ammonia, which is the nitrogen waste product from protein catabolism and a potent neurotoxin. It involves a series of biochemical steps in which nitrogen is removed from the blood and converted to urea, which is non-toxic and excreted in the urine. Urea cycle disorders (UCD) is a group of congenital disorders caused by enzyme or transporter deficiencies that impair the conversion of ammonia to urea. In UCDs, nitrogen removal is blocked, and nitrogen accumulates in the form of ammonia and other nitrogenous precursors of urea, causing acute episodes of hyperammonemia. The overall incidence of UCDs caused by enzyme deficiencies is approximately 1:8000 [1, 2] or newly 1:35,000 newborns [3]. UCDs include ornithine transcarbamylase deficiency (OTCD), the most common form; carbamoyl phosphate synthetase deficiency (CPSD); arginino-succinate synthetase deficiency (ASSD, citrullinemia type I); arginosuccinate lyase deficiency (ASLD, arginino-succinic aciduria); and more rarely, arginase deficiency (ARGD) and N-acetylglutamate synthetase deficiency (NAGSD). Hyperammonemia is also found in the hyperornithinemia-hyperammonemia-homocitrullinuria (HHH) syndrome caused by a mutation in the *SLC25A15* gene, which encodes the mitochondrial ornithine and citrulline transporter [4], and in lysinuric protein intolerance (LPI) caused by inherited recessive mutations in the *SLC7A7* gene, which encodes for the cationic amino-acids transporter subunit y + LAT1 located on the basolateral plasma membrane of epithelial cells and macrophages. Hyperammonemia can also be associated with other inherited metabolic diseases such as organic acidurias, mitochondrial fatty acid oxidation defects and Carbonic anhydrase VA (CAVA) deficiency, a new disease regulating carbamoyl phosphate synthetase 1, and with liver failure and certain cases of iatrogenic intoxication, e.g., those caused by sodium valproate [5].

If untreated, UCDs can cause increased levels of ammonia in the bloodstream (a normal level of plasma ammonium is < 100 μmol/L in neonates and < 50 μmol/L in children and adults), resulting in vomiting, lethargy, seizures, brain damage, coma and eventually, death. The first episode of hyperammoniemia may occur at any age, depending on the severity of the UC blockade. Neonatal coma is the most common presentation, with a risk of decompensation at any time throughout the lifespan, despite adequate medical and dietary treatment. Despite medical progress, UCDs remain at high risks of acute decompensation with neurological sequelae and vital risk [6], underlining the necessity of early diagnosis [7] and intensive consensual protocols to optimise neonatal but also long-term prognosis [2, 8–10].

The current treatment of UCD consists of a low-protein diet associated with medications that provide alternative pathways for the removal of nitrogenous precursors of urea or ammonia from the bloodstream [11]. Depending on the enzyme deficiency, these treatments must be combined with daily L-arginine and/or citrulline supplementation [12].

In cases of acute decompensation, and particularly in emergency situations caused by infection, the major complication of hyperammonemia is cerebral oedema [2, 13]. First-line emergency treatment depends on the clinical status and whether the oral route is available for drug administration. Treatment aims to normalize the plasma ammonium level and restore the nitrogen balance (to ensure that input = loss), stop protein intake, administrate emergency regimen with high lipid and carbohydrate intake, re-establish the excretion of excess nitrogen via the administration of ammonium ion scavengers (orally or via i.v.), and provide appropriate symptomatic management (rehydration, correction of electrolyte imbalance). If necessary, extracorporeal toxin removal procedures should be considered [14] (see below). After recovery and for the long term, a low-protein diet and oral ammonium ion scavengers should be prescribed.

Two active substances have been identified as good candidates to promote the removal of nitrogenous residues (and mainly ammonium ions) from the blood by acting as an ammonium ion trap [15]: i) Sodium benzoate conjugates with glycine to form hippurate, which is eliminated by the kidneys (one mole of sodium benzoate eliminates one mole of ammonia); ii) Sodium phenylacetate conjugates with glutamine to form phenylacetylglutamine, which is eliminated by the kidneys (one mole of sodium phenylacetate eliminates 2 moles of ammonia) [16]. Sodium or glycerol (not yet marketed in France) phenylbutyrate (per os), precursors of phenylacetate, also eliminates 2 moles of ammonia per mole.

For the daily treatment and prevention of hyperammonemia, sodium benzoate per os can be used with or without sodium or glycerol phenylbutyrate.

In cases of acute hyperammonemia [4, 17], emergency treatment in France consists of i) Sodium benzoate at a loading dose by i.v. route [18]. This treatment, ‘sodium benzoate AP-HP 1 g/10 mL solution for infusion’, is available as a hospital preparation. It is produced by the Pharmaceutical Establishment of the Paris Hospitals (Assistance Publique Hopitaux de Paris). Sodium phenylacetate can be combined with sodium benzoate (an equimolar mixture of both products). The combination of these two active substances is available in France on a named-patient prescription basis (ATU) [19]. It must be administered *via* a central venous catheter, which carries an increased risk of complications (infection, thrombosis) and the need for anaesthesia [20]. In cases of severe hyperammonemia (>400 μmol/L), extracorporeal toxin removal procedures [21] should be considered.

Despite the availability in France of the above-mentioned ammonium scavengers and a relatively consensual protocol for the treatment of acute episodes of hyperammonemia [22], there are no data specifically evaluating the efficacy of sodium benzoate for the treatment of hyperammonemia. The aim of this study was to evaluate the risk/benefit of providing i.v. sodium benzoate treatment at the onset of the acute hyperammonemic episodes and during the follow-up of UCD. We retrospectively studied patient files from all of the French reference centres specializing in the treatment of metabolic diseases where this drug is the reference treatment for acute episodes of hyperammoniemia.

## Methods

### Design

This was a retrospective multicentre study of the use of i.v. sodium benzoate in UCD patients hospitalized for an acute episode of decompensation with hyperammonemia at a French Reference Centre of Metabolic Diseases (*Filière Maladies Métaboliques*) between 2000 and 2010. Because this was a retrospective cohort study, no control group was deemed necessary. All hyperammonemia patients were treated with at least one dose of i.v. sodium benzoate.

The primary objective of this study was to describe the use of i.v. sodium benzoate for acute episodes of hyperammonemia in UCD patients, focusing on the justification of this treatment decision and the dosing schedule (rate of infusion, duration of infusion, total dose of i.v. sodium benzoate administered).

The secondary objective of this study was to describe the efficacy and safety of i.v. sodium benzoate for treating acute episodes of hyperammoniemia, especially in terms of the time course of ammonemia at admission, during hospitalization and at the end of the episode, and during the 24 h prior to discharge from hospital [16].

### Patients and treatments

The six reference centres enrolled in this retrospective study—Necker-Enfants Malades (AP-HP, Paris), Robert-Debré (AP-HP, Paris), Hospices Civils de Lyon (HCL), Jeanne-de-Flandre (Lille), La Timone (AP-HM, Marseille), and Brabois (Nancy) - were asked to retrieve the medical records of all patients with UCD who had been hospitalized between January 2000 and December 2010 in their centers, with a diagnosis of hyperammonemia (>100 μmol/L) or to minimize the likely risk of hyperammonemia and for whom emergency treatment with i.v. sodium benzoate was initiated.

Long-term treatment of patients included low-protein diet that was adapted to the tolerance of the individual combined with arginine and/or citrulline supplementation and sodium or glycerol phenylbutyrate and/or sodium benzoate administration, depending on the onset and severity of the disease. Emergency treatment with high-caloric intake providing glucose and lipids was performed during catabolic states.

The data from the records analysis have been collected in a secure electronic CRF (case report form), accessible with an individual login and password (database MySQL), developed by the Unité de Recherche Clinique (URC) of Hôpital Robert Debré, AP-HP. This database has been reported to the French computer watchdog authorities (Comité Consultatif pour le Traitement de l’Information en matière de Recherche pour la Santé, CCTIRS, and Commission Nationale Informatique et Liberté, CNIL). Records analysis and data monitoring based on medical charts were conducted by a clinical research associate who visited each centre over a one-year period.

For each episode, the following information was collected: triggering factors and initial and final biological values, especially the ammonemia time course during hospitalization. Other biological parameters included liver enzymes, clotting factors, blood pH, blood bicarbonates, serum bilirubin. Concomitant treatments were recorded. Adverse events (AEs) and deaths during hospitalization were also reported.

### Statistical analysis

Statistical analyses were performed using the R statistical software (http://cran.r-project.org/) in the Clinical Research Unit of Hôpital Necker, AP-HP. The data are provided for individuals and are essentially descriptive. Where relevant for the description of the cohort, quantitative data are expressed as the mean (± SD) or median (range), and qualitative data are expressed as counts and frequencies.

## Results

### Patients

Sixty-one patients who were hospitalized at the 6 Reference centres in France and required i.v. sodium benzoate were included in the study between November 2011 and August 2012 : 26/61(42.6 %) from Necker-AP-HP Paris, 16/61 (26.2 %) from HCL Lyon, 6/61 (9.8 %) from Robert Debré-AP-HP Paris, 6/61 (9.8 %) from AP-HM Marseille, 4/61 (6.6 %) from Lille, and 3/61 (4.9 %) from Nancy.

A total of 95 episodes were identified for these 61 patients: 38/95 (40.0 %) from Necker-AP-HP Paris, 28/95 (29.5 %) from HCL Lyon, 11/95 (11.6 %) from Robert Debré-AP-HP Paris, 10/95 (10.5 %) from AP-HM Marseille, 4/95 (4.2 %) from Lille, and 4/95 from Nancy (4.2 %).

The number of episodes per patient was 1 (*n =* 40: 68.6 %, mostly during the neonatal period), 2 (*n =* 14:23.0 %), and 3 (*n =* 4:6.6 %). Three patients underwent 4, 5 and 6 episodes of i.v. sodium benzoate treatment, respectively. Of these patients, two presented with OTCD and died, while the other one is still living with ASSD.

The patients’ ages ranged from 0 to 35 years, with a median age of 3.0 years. Over one-third of the patients (42.1 %) were between 2 and 11 years old (Table 1).

**Table 1:** Number of episodes by age category

Age category	Patients *N =* 61 (%)
0–27 days	25 (26.3 %)
1-23 months	14 (14.7 %)
2–11 years	40 (42.1 %)
12–17 years	10 (10.5 %)
≥18 years	6 (6.3 %)

The sex ratio of the patients in this study was 1:1 (50.8 % were female), although there was a slightly higher number of episodes in the male patients (54 episodes versus 41 in female patients).

Thirty-nine (53.4 %) of the episodes were initial episodes of neonatal coma.

### Enzyme deficiencies

OTCD was the most common enzyme deficiency (32.8 %, 20/61 patients – 10 males, 10 females), followed by ASSD (Table 2). Two patients had a suspicion of OTCD and one patient had a triple H syndrome. All of the patients except 2 (suspected of OTCD) had diagnostic confirmation at the molecular level.

**Table 2:** Causes of UCD-related hyperammonemia

Causes	Patients *N =* 61 (%)	Episodes *N =* 95 (%)
OTCD	20 (32.8 %)	35 (36.8 %)
ASSD	18 (29.5 %)	30 (31.6 %)
CPSD	15 (24.6 %)	18 (18.9 %)
ASLD	3 (4.9 %)	4 (4.2 %)
ARGD	1 (1.6 %)	2 (2.1 %)
NAGSD	1 (1.6 %)	2 (2.1 %)
Others	3 (4.9 %)*	4 (4.2 %)

*Suspected OTCD without a molecular basis *N =* 2, HHH syndrome *N =* 1

### Indication for i.v. sodium benzoate administration

The most frequent cause of decompensation reported in this study was infection (either benign or serious), with 20/36 episodes (55.5 %). The other causes were general anaesthesia (11/36, 30.6 %), excessive consumption of proteins (3/36, 8.3 %) and fasting (2/36, 5.6 %). Clear notification of the reason for the administration of i.v. sodium benzoate was not retrospectively found for the majority of the episodes (59/95 episodes, 62.1 %).

### Neurological status

The patients’ neurological state and clinical symptoms were recorded in the 24 h prior to the first infusion of sodium benzoate for each episode. Patients were in a coma for 28.1 % of episodes (25/89 recorded episodes) and had seizures for 18.6 % of episodes (16/86 recorded episodes). Vomiting was present for 40.0 % of episodes, fever for 13.7 % and diarrhoea for 9.5 % of episodes (*N =* 95 episodes).

### Doses of i.v. sodium benzoate administered

For 54 of the 95 episodes (56.8 %), the patients received their first dose of i.v. sodium benzoate in the intensive care unit, whilst for the other 41 episodes (43.2 %), the first dose of i.v. sodium benzoate was administered in other hospital departments.

A loading dose of i.v. sodium benzoate was given to patients for 41 episodes (*N =* 95 episodes, 43.2 %) using a peripheral line infusion. Precise dosage was missing for 6 of the episodes, but for the remaining 35 episodes, the median loading dose was 250.0 mg/kg [14–425].

Maintenance doses were given by peripheral i.v. infusion in most cases; a central i.v. line was used for only one episode. The maximum dose administered during the first period of treatment was missing for 21 of the episodes, but for the remaining 74 episodes, the median maximum dose was 246.1 mg/kg/day [25.7 –990.1].

The total treatment duration per episode was a median of 2 days [0.1 –13].

The total duration of hospitalization in the intensive care unit per episode (patients were admitted to the intensive care unit for 54 episodes) was a median of 4 days [0 –17], while the total duration of hospitalization per episode was a median of 10 days [0 –70].

### Efficacy

From a clinical point of view, the majority of the patients (69 %) had only one episode of hyperammoniemia; the median age of these patients was 3 years [0 –35 years]. The first dose of i.v. sodium benzoate was administered to patients upon admission in the intensive care unit of the hospital for 57 % of episodes and in other hospital departments for 43 % of episodes.

Eight patients died during hospitalization. The causes of death were the severe neonatal coma that revealed the disease (3 OTCD, 2 CPSD, 1 ASSD), decompensation during surgery (portal vein recanalization) at age 3.6 years (OTCD), and complications in the immediate course of liver transplantation at age 3.6 years (OTCD).

In terms of biological parameters the efficacy of i.v. sodium benzoate associated with emergency regimen was assessed by the decrease in ammonemia below the limit of 100 μmol/L by the end of i.v. sodium benzoate treatment.

The ammonia results at the end of i.v. treatment were missing for 18/95 episodes of this cohort; however after these episodes, the corresponding patients were successfully discharged from hospital, implying that their ammonia levels had returned to normal (or at least to non-toxic) levels.

Of the 77/95 documented episodes, the median ammonia level before i.v. sodium benzoate treatment was 245.5 μmol/L [20.0; 2274.0]. This median ammonemia level decreased to 40.0 μmol/L [13.0; 181.0] by the end of benzoate treatment. When we restricted the episodes to those with ammonemia level before treatment > 100 μmol/L (69 episodes), the median ammonemia level before i.v. sodium benzoate treatment was 291 μmol/L [101 –2274]. This median ammonemia level decreased to 41 μmol/L [13 –181] by the end of benzoate treatment. The median degree of ammonemia at discharge from hospital was 41 μmol/L [28.8; 48.0]. More precisely, the treatment was very successful (ammonemia ≤ 100 μmol/L) for 64/69 of the episodes recorded for the 53 surviving patients/61 (92.8 %).

Ammonemia was still > 100 μmol/L at the end of i.v. treatment for 5 episodes; however, these 5 patients were successfully discharged from hospital, 4 with ammonemia ≤ 100 μmol/L at discharge (the results were missing for one episode). These 5 patients received another treatment for hyperammonemia at the end of their i.v. sodium benzoate treatment (neonatal comas treated with the combination of sodium phenylacetate and sodium benzoate + haemofiltration or the combination of sodium phenylacetate and sodium benzoate alone).

### Other treatments

Patients were treated with the combination of sodium phenylacetate and sodium benzoate during 10 of the episodes (*N =* 94 episodes, one observation missing; Table 3); for 6 of these episodes, the drug was given at the end of the treatment with i.v. sodium benzoate.

**Table 3:** Other NH3 treatments provided at the end of treatment with i.v. sodium benzoate (Emergency regimen was performed in all cases)

Treatments	Episodes *N =* 95 (%)
Missing	12 (12.6 %)
Sodium benzoate p.o.	65 (78.3 %)^a^
Sodium benzoate p.o. + Phenylbutyrate p.o.	3 (3.6 %)^a^
Combination Sodium phenylacetate + Sodium benzoate (central i.v. infusion)	3 (3.6 %)^a^
Combination Sodium phenylacetate + Sodium benzoate (central i.v. infusion) + Haemofiltration	3 (3.6 %)^a^
Haemofiltration	2 (2.4 %)^a^
Haemofiltration + Phenylbutyrate p.o.	1 (1.2 %)^a^
Phenylbutyrate p.o.	1 (1.2 %)^a^

^a^Calculated as a percentage of the non-missing data

Fifteen patients were treated with haemofiltration during 16 episodes; for 6 of these episodes, haemofiltration was provided at the end of treatment with i.v. sodium benzoate.

Patients were treated with antibiotics during 43 of the episodes (45.7 %, *N =* 94 episodes, one observation missing).

At the end of treatment with i.v. sodium benzoate, the most common treatment was the oral form of sodium benzoate (sodium benzoate p.o., 78.3 %, 65/83 recorded episodes). For 2/83 recorded episodes (2.4 %), no further treatment was administered.

### Safety

In this study, eighteen complications were related to the infusion (extravasation of the venous catheter, local oedema, local erythema, haematoma, local inflammation, and local abscess).

Because of metabolic decompensation, neurological signs were described in 19 episodes; liver symptoms were noted in 22 episodes (10 liver failures and 12 cases of liver cytolysis without liver failure). Also noted were 4 episodes of disseminated intravascular coagulation, 3 kidney failure episodes, one case of sepsis of the internal jugular vein catheter with septic shock, one case of left ventricular dysfunction, and one cardiorespiratory arrest lasting 4 min.

## Discussion

Sodium benzoate is an established and accepted adjunctive therapy for UCDs, as mentioned in the European guidelines [10], and for all causes of hyperammoniemia. Clinicians have used i.v.sodium benzoate routinely from the past 25 years to treat hyperammonemia in situations of acute decompensation (hyperammonemia) and during fasting, such as in cases of surgery or vomiting. Its efficacy and safety have been well known for years by metabolic physicians. Moreover, as previously reported (22), the most frequent cause of metabolic decompensation in our study was a catabolism episode, such as an infection. This underlines assertions that the availability of intravenous sodium benzoate is crucial for the management of UCD patients [23].

However, i.v. sodium benzoate has no marketing authorisation, and very little information on the use of this drug has been published. This retrospective study was established in 2011–2012 to consolidate the French physicians’ previous clinical experiences in the treatment of hyperammonemia in recent years. Data from 61 patients and 95 episodes of hyperammonemia were analysed during this study. These data showed that i.v. sodium benzoate decreased ammonia levels to ≤ 100 μmol/L by the end of treatment in most cases of late decompensation and that normal levels of NH3 were maintained in other situations where this treatment was prescribed to prevent decompensation. Also, this treatment is used for the prevention of decompensations and for the treatment of overt decompensations. Before treatment, the median ammonemia level was 246 μmol/L whatever the situations (overt decompensation or prevention of decompensation) (range: 20–2274 μmol/L, *N =* 82 episodes); it decreased to 40 μmol/L (range: 13–181 μmol/L, *N =* 77 episodes) among the patients who were still alive at the end of treatment with i.v. sodium benzoate. Deaths were caused by the underlying metabolic disease and occurred during the neonatal coma in 6 cases and during surgery/liver transplantation in 2 patients. Our study also confirmed that OTCD and ASSD are the most frequent UCDs (33 % and 30 %, respectively), while OTCD and CPSD are the most severe (representing all deaths in our study). It is well established that deaths occur mainly in severe neonatal coma, usually caused by OTCD and CPSD.

Because the first dose of i.v. sodium benzoate may be administered to patients both in the intensive care unit of the hospital than in other hospital departments, it is important for local hospitals to have i.v. sodium benzoate readily available before transferring a patient to a reference centre. Importantly, our work described the use of i.v. sodium benzoate in patients hospitalized in the French reference centers only. It is not representative of all episodes of hospitalizations and decompensations of the patients who firstly go to their nearby hospital, with telephone advices and emergency certificates from their reference center. Moreover initial comas are not managed only in reference centers as to date a lot of hospitals perform haemodialysis and acute medical treatments. All these considerations reinforce the necessity to have i.v. medications readily available in the whole national territory. The use of nitrogen scavengers is crucial, but of course associated with an intensive care management including rehydration, induction of anabolism with adequate amounts of calories, administration of i.v. arginine, carglumic acid for NAGS, CAVA and sometimes CPS1 deficiencies.

In our cohort, eighteen side effects were related to the infusion (extravasation of the venous catheter, local oedema, inflammation or abscess, haematoma). They were probably under-estimated in this retrospective study, but importantly no severe side effects were attributed specifically to i.v. sodium benzoate. Serious complications like comas, death and liver failure have been attributed to the underlying UCD and its decompensations.

A loading dose of i.v. sodium benzoate was given for 43 % of episodes, and the dose was variable. The fact that there were no guidelines available for most of the study period probably contributed to the lack of consistency in the administration and reporting of loading doses. Maintenance doses were given via peripheral i.v. infusion in most cases; central i.v. infusion was used for only one episode. The median maximum dose was 246.1 mg/kg/day, in accordance with the guidelines [10].

The total duration of i.v. sodium benzoate treatment per episode was a median of 2 days [0.1 –13], patients stayed in the intensive care unit for a median of 4 days, and the total length of stay in hospital was a median of 10 days. This point is important to note: not only the peak of plasma ammonium level, but also the duration of hyperammoniemia until normalization, have an impact on the outcome. More precisely, ammonemia was still > 100 μmol/L at the end of i.v. treatment for only 5 episodes. However, the 5 patients were successfully discharged from hospital (4 with ammonemia ≤ 100 μmol/L at discharge, the results were missing for one episode).

During 10 episodes, patients were treated with the combination of sodium phenylacetate and sodium benzoate (administered via central i.v. infusion) in association with i.v. sodium benzoate. This underlines the interest in i.v. sodium benzoate, which only requires peripheral vein access. Haemofiltration was required for 16 episodes, mostly in cases of neonatal coma for which i.v. sodium benzoate was not sufficient.

In our study, i.v. benzoate was switched to the oral form of sodium benzoate (sodium benzoate p.o., 78.3 %, 65/83 recorded episodes).

Whilst data were being collected for this French study, Häberle et al. published guidelines with the aim of harmonising the diagnosis and management of UCD in Europe [10]. These guidelines pool the results of and experience with all UCD treatments from several European countries, with most of the evidence based on expert opinion or case studies because of a lack of formal clinical trial data in Europe. The guidelines state that the ammonia scavengers sodium benzoate and sodium phenylacetate (one of the 2 active ingredients in the combination of sodium phenylacetate and sodium benzoate, along with sodium benzoate) are among the main treatments for acute hyperammonemia. The dose of i.v. sodium benzoate quoted in the guidelines is the same as that suggested in the product information sheet provided by AGEPS, AP-HP, which summarizes the doses of sodium benzoate AP-HP 1 g/10 mL to be used in clinical practice in France.

The experiences of the French clinicians indicated that i.v. sodium benzoate has advantages over the other treatments mentioned above. First of all, it can be administered during a coma or in cases of gastroenteritis, whereas the p.o. drugs containing sodium or glycerol phenylbutyrate cannot be used or are not well absorbed; and secondly, it is administered *via* a peripheral venous catheter, which is thought to be less risky [16, 20] and more practical than administration via a central venous catheter, as is the required for the combination of sodium phenylacetate and sodium benzoate. Another advantage concerns the availability of the products: i.v. sodium benzoate is available in France with a hospital preparation status, whereas the combination of sodium phenylacetate and sodium benzoate has to be imported on a named-patient basis from the USA (it has temporary use agreement —ATU - status in France). These advantages make i.v. sodium benzoate an essential tool in the treatment of hyperammoniemia, and all hospitals should own this treatment, particularly to treat the patient during the transfer to a specialized hospital. Importantly, secondary care centres and local hospitals may need to treat hyperammonemic decompensations related to UCD, as reference centres may have difficulty managing all UCD patients.

## Conclusion

The 10-year retrospective study from 2000 until 2010 in the 6 French reference centrers for metabolic diseases shows that i.v. sodium benzoate treatment is effective for the treatment and/or the prevention of hyperammonemia in UCD. This study also confirms the safety of this treatment. I.v. sodium benzoate is the only peripheral treatment recommended and used in France in acute episodes of UCD, associated with an emergency regimen. Consequently, this precious i.v. treatment should be readily available in emergency in all hospitals.

## Abbreviations

AE: Adverse event
AGEPS: Agence generale des equipements et produits de santé
ALT: Alanine aminotransferase
AP-HM: Assistance publique-hôpitaux de Marseille
AP-HP: Assistance publique-hopitaux de Paris
ARGD: Arginase deficiency
ASLD: Arginosuccinate lyase deficiency (arginino-succinic aciduria)
ASSD: Arginosuccinate synthetase deficiency (citrullinemia type I)
AST: Aspartate aminotransferase
ATU: Autorisation temporaire d’utilisation (authorisation for temporary use)
CCTIRS: Comité consultatif sur le Traitement de l’Information en matière de Recherche dans le domaine de la Santé
CHRU: Centre hospitalier régional universitaire
CHU: Centre hospitalier universitaire
CIC: Centre d’investigation clinique
CNIL: Commission nationale de l’informatique et des Libertés
CPSD: Carbamoyl phosphate synthetase deficiency
HCL: Hospices civils de Lyon
HHH: Hyperornithinemia-hyperammonemia-homocitrullinuria
i.v.: Intravenous
LPI: Lysinuric protein intolerance
NAGSD: N-acetyl-glutamate synthetase deficiency
OTCD: Ornithine transcarbamylase deficiency
p.o.: Per os
Q1: Lower quartile
Q3: Upper quartile
SD: Standard deviation
UC: Urea cycle
UCD: Urea cycle disorder
URC: Clinical research Unit
